# Paper-Based Colorimetric pH Test Strip Using Bio-Derived Dyes

**DOI:** 10.3390/bios15120816

**Published:** 2025-12-16

**Authors:** Aramis A. Sánchez, Darwin Castillo, Grettel Riofrío-Cabrera, Greysy Jaramillo, Vasudevan Lakshminarayanan

**Affiliations:** 1 PROSUR Construcción Sustentable del Sur, Puebla 72810, Mexico; 2 Department of Chemistry, Universidad Técnica Particular de Loja, Loja 1101608, Ecuador; gariofrio1@utpl.edu.ec (G.R.-C.); gmjaramillo2@utpl.edu.ec (G.J.); 3 Theoretical and Experimental Epistemology Lab, School of Optometry and Vision Science, University of Waterloo, Waterloo, ON N2L3G1, Canada; vengulak@uwaterloo.ca; 4 Departments of Physics, Electrical and Computer Engineering, and Systems Design Engineering, University of Waterloo, Waterloo, ON N2L3G1, Canada

**Keywords:** natural dyes, pH, testing strip, pigments, dyes, environment, cabbage, radish, turmeric, sensor

## Abstract

Natural dyes have emerged as a promising alternative to synthetic dyes for industrial applications due to their advantages, namely, easy availability, low cost, and environmental friendliness. In this sense, natural dyes, due to their potential to react over the pH range, could offer an alternative to conventional pH measuring techniques for industrial products, such as potentiometers, sensors, or indicator drops. Therefore, this project aims to evaluate the potential of several natural organic dyes in response to changes in pH and develop an indicator for determining pH grades. We extracted and analyzed the pigments of forty natural vegetable species using two extraction methods with a mixture of solvents, specifically 70% MeOH/30% H_2_O. The results find that pigments of *cabbage*, *hibiscus flower*, *radish*, and *turmeric* in their dry state exhibit the best reaction over a broad pH range, and color can be easily distinguished according to its level. These findings demonstrate the potential of natural dyes as a novel approach for pH verification, providing a sustainable and cost-effective alternative to conventional techniques.

## 1. Introduction

The measurement of pH plays an important role in understanding chemical reactivity, solubility, and the structural behavior of compounds, as well as in ensuring the proper functioning of biological systems [1,2]. From industrial processes and food biotechnology to environmental and clinical analysis, pH monitoring remains a critical parameter in both research and applied contexts.

Conventional pH meters based on electrochemical glass electrodes offer high precision but can be expensive, require calibration, and are often impractical in low-resource or field settings [3]. Synthetic acid–base indicators are another standard tool, but they raise concerns regarding safety and environmental impact due to their toxicity and lack of biodegradability [4]. Traditional synthetic dyes, such as phenolphthalein, methyl red, and bromothymol blue, although favored for their intense color and sharp responses in volumetric analysis, present significant drawbacks, including potential toxicity, environmental persistence, and restrictions on their use in food or consumer contact applications [5,6]. For example, phenolphthalein has been implicated in intensifying carcinogenic processes and causing alterations in the p53 gene [5]. This necessity has motivated intensive research for safer, more sustainable pH-sensing systems aligned with the principles of green analytical chemistry [7].

In this context, plant-derived natural dyes have emerged as compelling candidates to replace or complement conventional indicators [8,9]. These pigments are typically abundant, inexpensive, and biodegradable, and they are generally regarded as safe, making them attractive for developing disposable sensors in regions with limited resources or strict environmental regulations [10,11].

Natural colorants, especially anthocyanins [12], betalains [13], curcuminoids [14], flavones, and carotenoids, exhibit chromophoric properties that respond sensitively to pH changes and can be recovered from agricultural products or biowaste [5], such as red cabbage [15], berries, beetroot, turmeric [16], and hibiscus flowers [9,17].

The functional mechanism of many of these pigments, especially anthocyanins [12], is based on reversible structural transformations of the flavylium cation into quinoidal bases or chalcone forms as the hydrogen ion concentration changes, resulting in visible color shifts over specific pH ranges [18]. Beyond their pH responsiveness, many natural pigments are bioactive (e.g., antioxidants, antimicrobials), which has spurred their integration into intelligent packaging [10,19,20], optical sensors, and other functional materials, such as solar cells [21] and organic LEDs [22].

A growing body of work has demonstrated that plant extracts can serve as effective acid-base indicators in classical titrations [7,23]. For example, Raghavendra et al. [5] utilized plant biowaste to obtain green acid–base indicators, exploring their phytochemical profiles and quantum-chemical properties. In other work, they evaluated beetroot and pomegranate peel extracts as titration indicators with antioxidant activity [6]. The extracts from fig leaves (*Ficus carica*), pomegranate leaves (*Punica granatum*), and Mussaenda flowers (*Mussaenda philippica*) have been reported to yield titration endpoints that coincide or closely agree with those obtained using phenolphthalein, often with low percentage errors on the order of 1.6–4.5% [5]. Ghatage et al. systematically compared pigment extracts as pH indicators in titrimetric analysis [7], while Korfii et al. [24] demonstrated that red mangrove extracts can serve as practical pH indicators in aqueous systems.

A secondary data analysis from Tuslinah et al. [18] of ethanol extracts from anthocyanin-containing plants such as Adam’s Eve leaves (*Rheo discolor*), white frangipani flowers, and Telang flowers (*Clitoria teratea* L.) found no statistically significant differences in endpoint pH detection compared with phenolphthalein (*p* > 0.05), with relative standard deviations typically ≤1–2%, indicating high precision under the tested conditions. These results support the analytical viability of plant-based dyes as pH indicators, providing robust and low-cost pH indication in liquid assays. However, their implementation as standardized, ready-to-use test strips remains less explored.

Natural pigments have also been explored in more application-oriented formats. Specific applications of natural dyes as indicators are also being reported in food quality control [25]. For example, Sha et al. [23] validated aqueous extracts of *Ruellia* simplex flowers as indicators for milk quality assessment, demonstrating high accuracy and excellent linearity (R^2^ ≈ 0.978) comparable to established dye reduction tests, and highlighting the potential of plant-based colorants as field-ready analytical tools in dairy diagnostics.

Wu et al. [15] include red-cabbage puree/poly (vinyl alcohol) films for monitoring fish freshness; Ma et al. [26] developed pH-sensitive chitosan quaternary ammonium films loaded with anthocyanins for pork; Mohseni-Shahri and Moeinpour [27] explored gelatin films co-loaded with curcumin and anthocyanins for shrimp; and Faisal et al. [28] developed composite films that combine anthocyanins with the synthetic dye neutral red to expand the dynamic response range. These systems typically offer improved handling, mechanical robustness, and reduced dye leaching compared with simple liquid indicators.

On the other hand, films and hydrogels containing synthetic pH-sensitive dyes or conducting polymers such as polyaniline (PANI) have been developed for optical readout using simple LED-based devices, illustrating how low-cost optical transduction can complement colorimetric materials in real-time pH monitoring [29]. Overall, these studies show that natural extracts can, at least in certain contexts, match traditional synthetic indicators in terms of accuracy, sensitivity, and precision, while also reducing toxicity and enabling integration into biodegradable materials.

In parallel, paper-based analytical devices (PADs) have emerged as attractive platforms for low-cost sensing, enabling capillary-driven assays and optical readout with minimal equipment [30]. Most PADs reported to date incorporate synthetic dyes or electroactive materials, such as polyaniline (PANI)-based optical pH layers [29], which offer good sensitivity but rely on petrochemical components and may raise environmental or safety concerns. There is, therefore, a growing interest in PADs that use fully bio-derived colorants while retaining quantitative or semi-quantitative performance.

Despite these advances, important challenges remain. Natural pigment sensors frequently exhibit limited stability: anthocyanins and related compounds may degrade or lose color intensity under prolonged exposure to high pH, light, oxygen, or elevated temperature, which can shorten the practical shelf life of test strips and films [12,31]. Ensuring consistent calibration of color changes to precise pH values is also non-trivial, as perceived color is influenced by ambient lighting, dye loading, substrate properties, and the optical characteristics of the imaging system. Moreover, reproducibility can be problematic: differences in plant source, harvest conditions, extraction protocol, or storage can alter the composition of multicomponent extracts, leading to sensor-to-sensor variability in color response [5,11,32]. The secondary analysis of natural dyes [18] versus phenolphthalein further illustrates that, while precision and apparent equivalence can be demonstrated in specific titration settings, key aspects such as long-term storage stability, effects of ionic strength and interfering substances, lighting requirements, and user training remain insufficiently characterized. These gaps underscore the need for systematic studies that not only explore new dye sources but also explicitly address stability, calibration, and reproducibility under practical conditions.

In this context, the present work aims to develop and characterize a simple, paper-based pH test strip utilizing pigments extracted from natural vegetable dyes. We screened forty plant species readily available in the Ecuadorian market, selected based on accessibility and non-endangered status, to identify those with the clearest and broadest pH-responsive color transitions. From this survey, four bio-derived dyes—*hibiscus*, *purple cabbage*, *radish*, and *turmeric*—were chosen for detailed study based on their distinct color changes over the pH scale. The corresponding extracts were immobilized on mixed cotton–cellulose pads to construct multi-zone paper strips that, in combination, visually cover the full pH range from 0 to 14 using only natural pigments. We then characterized their response to standard buffer solutions under controlled illumination, documented the resulting color matrices, and evaluated short-term stability in both liquid and dry formats over storage periods of 15–30 days.

In addition to visual inspection, we explored a deliberately low-complexity grayscale-based digital image analysis as a semi-quantitative readout, using region-of-interest averaging to mitigate local non-uniformities in dye deposition. This choice was made to approximate a realistic, resource-limited scenario in which only basic camera hardware and straightforward processing are available, while acknowledging that more sophisticated RGB/HSV workflows could further improve precision in future work. Throughout the manuscript, we explicitly frame the system as a bio-derived, semi-quantitative pH test strip and discuss its limitations in stability, calibration, and reproducibility, as well as clear directions for optimization (e.g., long-term aging studies, interference testing, and comparison across multiple harvests and extraction batches). Overall, this study presents a sustainable, low-cost proof-of-concept for biodegradable pH test strips based on natural dyes, with potential applications in educational settings, on-site environmental monitoring, and quality control in food and cosmetic products.

## 2. Materials and Methods

### 2.1. Selection of Plant Sources

The experimental process began with the selection of 40 plant species readily available in the Ecuadorian market. The criteria for selection were (1) non-endangered status, ensuring compliance with environmental and conservation regulations, and (2) accessibility in local markets to ensure potential reproducibility and scalability. The selected species included a variety of edible fruits, vegetables, flowers, and spices traditionally used for their natural pigmentation (see Table 1).

### 2.2. Extraction of Natural Pigments

In the present study, all extracts were prepared from plant material acquired in a single region and time period, using a standardized maceration and drying protocol. We did not perform a systematic comparison of different harvests or geographical batches, nor did we conduct chromatographic quantification of individual pigments. Therefore, our data reflect a proof of concept for one set of extract pigments. This research opens the door to the possibility of creating controlled crops if the process is to be industrialized, thereby guaranteeing the colorimetric characteristics of the pigment and eliminating differences that can occur due to various factors, such as geographical origin, climate, and harvesting conditions.

Pigments were extracted using a dynamic-static maceration protocol. Initially, dynamic maceration was performed for 1 hour under continuous agitation to enhance cell wall disruption and pigment release. This step was followed by static maceration for 23 h at room temperature, allowing for the complete diffusion of the pigments into the solvent.

The extraction solvent used was a binary mixture of 70% methanol and 30% distilled water (*v*/*v*), chosen for its efficiency in extracting both hydrophilic and moderately lipophilic compounds. A solid-to-solvent ratio of 1:10 (*w*/*v*) was maintained to standardize extraction yields across different samples. Although methanol is a toxic solvent and not ideal per green chemistry principles, its use was justified by the high extraction efficiency for a broad range of pigment compounds; moreover, the solvent was completely evaporated after extraction, minimizing its presence in the final product. All extractions were performed with proper safety measures (ventilation, PPE), and the dried pigment extracts contained no residual methanol. In future developments, greener solvent alternatives (e.g., ethanol or water-based systems) will be explored to further improve the eco-friendliness of the process.

After maceration, filtration was performed using vacuum filtration (simply accelerates the process by generating a pressure difference across the filter funnel) and qualitative filter paper (WHATMAN CYTIVA 1440 Grade 40 quantitative cellulose filter paper, 8 µm, Corona, CA, USA) to remove residual plant solids. The resulting filtrate contained the dissolved pigment extract.

**Figure 1:**
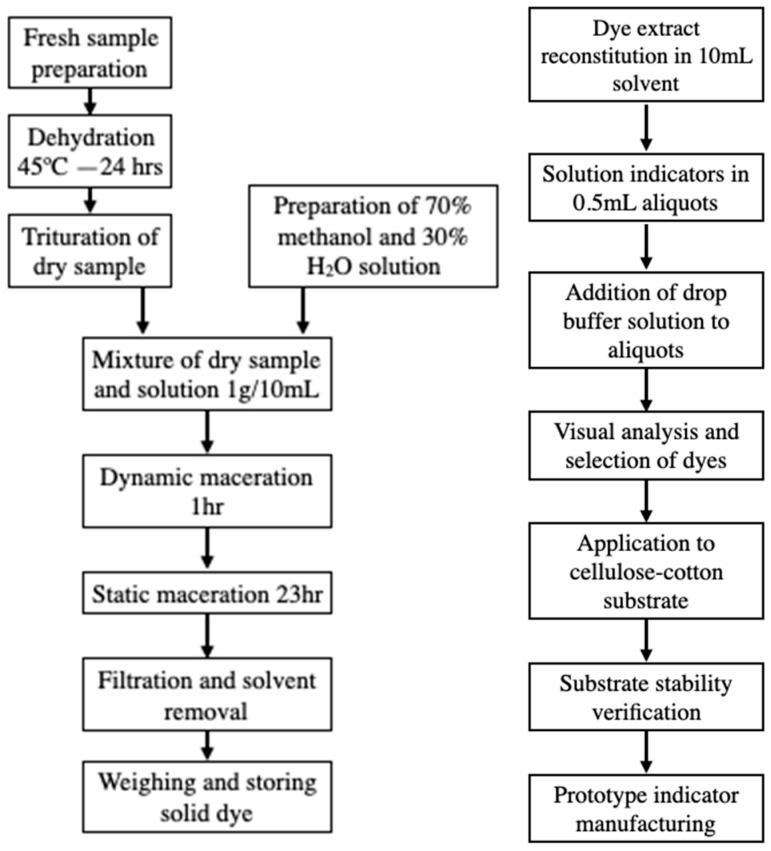
(**Left**) Diagram of dye extraction and filtration. (**Right**) Diagram of experimental testing and prototype manufacturing.

To obtain the concentrated colorant, the filtrate was evaporated using a rotary evaporator (model RE-2S-VD, LABFREEZ, Beijing, China) under reduced pressure at 40 °C until a dry pigment concentrate was obtained. This step was crucial for standardizing pigment dosage in subsequent tests and calculating extraction yield per plant material (see Figure 1).

### 2.3. Preparation of Indicator Solutions

Each pigment extract was reconstituted by dissolving an aliquot of the dried pigment in 10 mL of distilled water, forming a stock solution. From this solution, 14 aliquots of 0.5 mL were dispensed into transparent glass vials, one for each of the 14 buffer pH values.

To each vial, a single drop of buffer solution at a defined pH was added. The buffer solutions spanned a pH range of 1 to 14, prepared using standard analytical-grade buffer reagents. For the most acidic point (pH 0), a diluted hydrochloric acid solution (0.1 M HCl) was used to approximate a pH 0 buffer, extending the range for later tests. The pH of each buffer was verified using a calibrated pH meter (AB 200 ACCUMET, Fisher, Madrid, Spain) to ensure accuracy.

**Figure 2:**
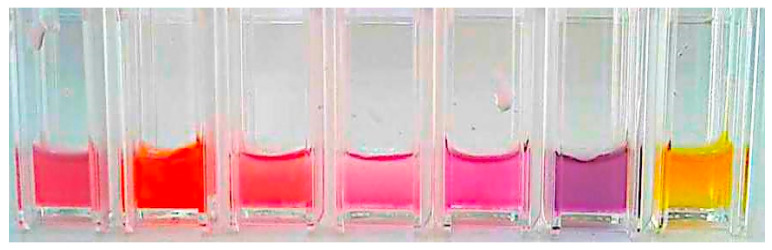
Radish peel dye in 0.5 mL vials exposed to pH levels of 1, 3, 5, 7, 9, 11, and 13, from left to right.

A visual inspection was then carried out under standardized lighting conditions, using a white background and a cold white LED light (BR30, 65W, Sunco, CA, USA) from the same distance and position, to evaluate chromatic transitions. Pigments showing clearly distinguishable color changes across multiple pH levels were considered as preselected candidates (see Figure 2).

### 2.4. Application to Paper Substrate

**Figure 3:**
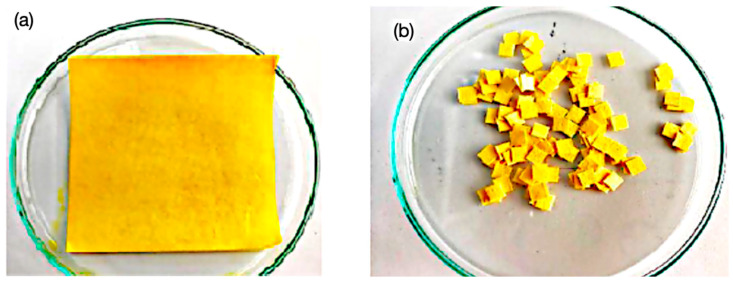
(**a**) Turmeric dye used to impregnate the paper substrate, 10 × 10 cm, 50% cotton and 50% cellulose. (**b**) Impregnated paper substrate in 5 × 5 mm samples ready to be mounted on acetate paper strips.

The preselected pigments were further evaluated for stability and responsiveness when applied to a cellulose-cotton matrix. The substrate used was a blend of 50% cellulose and 50% cotton paper, cut into uniform strips and 5 × 5 mm square pads (see Figure 3). Pigment solutions were applied to the paper pads by either immersing the pads in the solution or drop-casting the solution onto the pad until it was fully saturated and then allowing it to dry under ambient conditions. Drying was performed in the dark and at room temperature (avoiding direct sunlight and excessive humidity) to preserve pigment integrity. After drying, each colored paper pad (5 mm × 5 mm) was affixed onto a plastic support strip (transparent acetate, Hygloss, NJ, USA) using a neutral-cure silicone adhesive (SP-0510, Sayer, Mexico). The silicone was applied in a very small amount at the edge of the pad to minimize contact with the pigmented area. Importantly, this silicone adhesive was tested to ensure it had no intrinsic acidity or alkalinity that could influence the indicator (it showed neutral pH and caused no color change in an undyed paper control). The adhesive was allowed to cure for 24 h, yielding assembled test strips with the pigment pads firmly attached.

**Figure 4:**
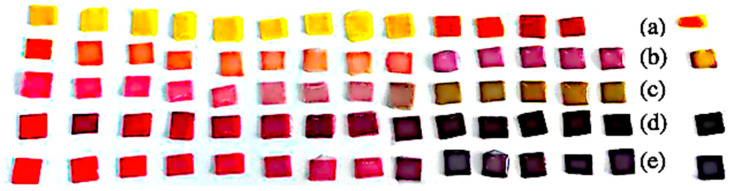
Side-by-side photograph of paper-based pH test pads for five dye systems (rows (**a**–**e**)). Rows (**a**–**e**) correspond to five distinct natural dye extracts (from top to bottom): *turmeric*, *radish*, *purple cabbage pigment* extracted with water, hibiscus, and purple cabbage. Within each row, pads are ordered from lower to higher pH (left to right), illustrating the changes in hue and intensity across the selected pH set.

Pigment solutions were applied to the paper strips by immersion or drop-casting and then dried under ambient conditions, avoiding direct sunlight and excessive humidity. Once dry, the strips were exposed again to standard buffer solutions across the entire pH range to determine if their chromatic response was preserved in a solid-state format (see Figure 4).

The selection criteria at this stage included the following:

- Color contrast between pH values.
- Reversibility of the color response.

Stability of the strips under storage in dry and dark conditions for up to 30 days.

### 2.5. Digital Image Analysis

To evaluate the analytical potential of the selected indicators, the colored paper strips were subjected to image-based analysis using grayscale intensity profiling. High-resolution photographs (12 MP, f/2.8, Apple iPhone 11, Apple Inc., Cupertino, CA, USA) were taken under controlled lighting and background conditions, and the image data were processed using open-source software, such as ImageJ (ImageJ version 154, NIH, Bethesda, MD, USA) [33].

**Figure 5:**
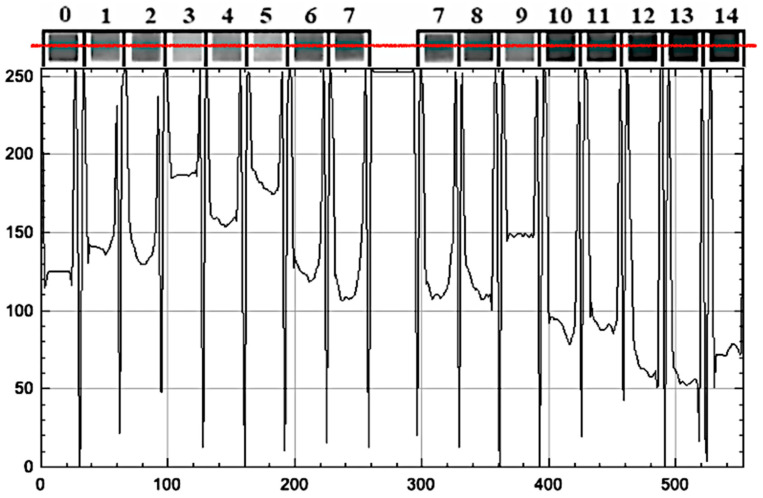
Grayscale intensity (0–255) along a horizontal scan (red line) of a radish-dye pH strip, with pad boundaries and pH labels (0–14) indicated; peak–trough patterns reflect pH-dependent contrast.

The color response of each strip to pH was analyzed based on its grayscale intensity profile, enabling a semi-quantitative comparison between samples. This analysis aimed to determine whether the colorimetric variation could be digitally interpreted and correlated to specific pH values, enabling future integration into electronic pH sensing systems. This produced intensity peaks and troughs corresponding to each pad’s position and darkness. However, given that the dye distribution on some pads was not perfectly uniform, the analysis method was refined by sampling multiple points across each pad area. Specifically, we selected three representative regions (center and many opposite side points) on each pad and recorded the grayscale intensity in those regions (see Figure 5), then averaged these values. This multi-point sampling approach provides an average grayscale intensity for the entire pad, thereby mitigating the influence of uneven color distribution or edge effects.

### 2.6. Final Indicator Selection and Prototype Design

Based on the cumulative results, the four most promising pigments were selected for the development of a prototype. These were chosen for the following qualities:

- Strong and distinguishable chromatic transition across wide pH ranges.
- Adequate stability on paper matrices.
- Clear grayscale profiles suitable for image-based detection.

The four selected pigments were *Hibiscus sabdariffa* (roselle flower), *Brassica oleracea* var. *capitata f. rubra* (purple cabbage), *Raphanus sativus* (red radish), and *Curcuma longa* (turmeric). Notably, the first three are anthocyanin-rich extracts, while the last is a curcuminoid-based extract, representing two major classes of natural pH-sensitive pigments.

The final step involved fabricating pH indicator test strips using the selected pigments. These strips were prepared in standardized dimensions and tested for practical usability in laboratory and field conditions.

## 3. Results

### 3.1. Visual Response to pH in Preselected Pigments

From the initial screening of 40 plant-based extracts, four pigments were selected based on their strong and distinguishable chromatic response across a broad range of pH values (see Table 2):

1. *Hibiscus sabdariffa* (Hibiscus flower)
2. *Brassica oleracea* var. *capitata f. rubra* (Purple cabbage)
3. *Raphanus sativus* (Radish)
4. *Curcuma longa* (Turmeric)

**Figure 6:**
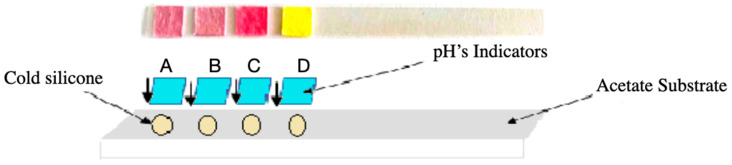
Assembly of the acetate-based test strip, showing the four dye-impregnated pads (A–D) mounted with cold silicone on the acetate substrate prior to pH exposure.

Each pigment was tested both in aqueous solution and embedded into a solid matrix of paper (50% cellulose, 50% cotton) cut into 5 mm × 5 mm squares. These were mounted onto acetate strips using cold silicone glue (carefully chosen to avoid any alteration with the change in pH of the solutions) to avoid altering the colorant’s stability and chemical behavior (see Figure 6). After drying, the indicator strips were exposed to buffer solutions with pH values ranging from 0 to 14.

### 3.2. Qualitative Visual Evaluation

**Figure 7:**
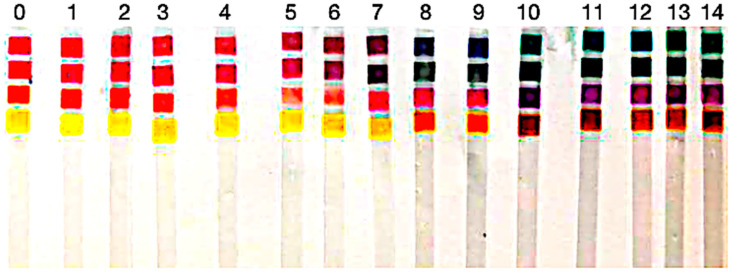
Photograph of paper strips dyed with the four bio-derived colorants after exposure to buffer solutions from pH 0–14 under controlled illumination. Columns are ordered left-to-right by pH; the labels above indicate the buffer value for each column, and the stacked pads within each column show the dye-dependent color response. The rows (colorant dye) from Table 2 correspond to the rows in this figure.

Upon exposure to the buffer solutions, the samples displayed visually detectable color transitions over different pH ranges. This color shift was immediate for most indicators, particularly in acidic or strongly alkaline conditions (see Figure 7).

This visual assessment highlights that the dyes stand out as pigments capable of demonstrating detectable color changes throughout the entire pH spectrum (0–14). In contrast, other pigments displayed limitations in very acidic environments (pH < 3).

Notably, visual inspection remains the most reliable and practical method for determining pH ranges with these natural indicators. While digital processing offers quantitative insights, human vision is still superior in recognizing subtle color gradients across a spectrum, particularly in low-resolution or uneven lighting conditions.

Under uncalibrated conditions, visual perception remains the gold standard for practical pH estimation using natural pigments, particularly when employed outside laboratory settings.

### 3.3. Digital Image Analysis and Grayscale Profiling

To explore the potential for quantitative digital pH estimation, all samples were photographed under standardized illumination on a white background and using a cold white LED light at a consistent distance. The digital images were then converted to grayscale, and intensity profiles were extracted for each pH condition using image analysis software (ImageJ).

**Figure 8:**
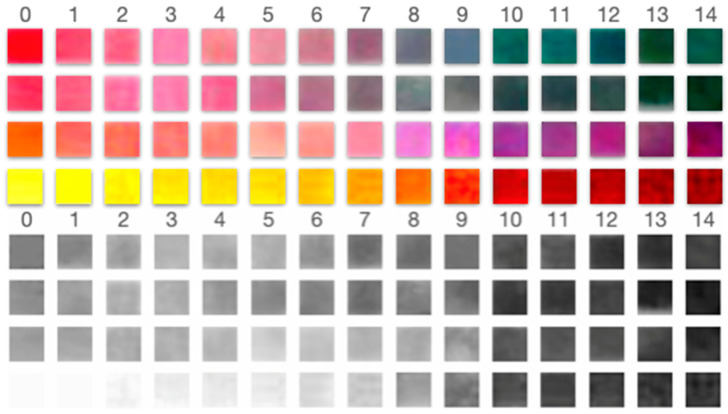
Color and grayscale matrices for the four dye systems across buffer pH 0–14. Top panels: Original color pads ordered from low to high pH (left to right). Bottom panels: corresponding grayscale renderings used for intensity analysis. These matrices illustrate the pH-dependent hue/brightness shifts that underpin the grayscale detectability reported in Table 3.

**Figure 9:**
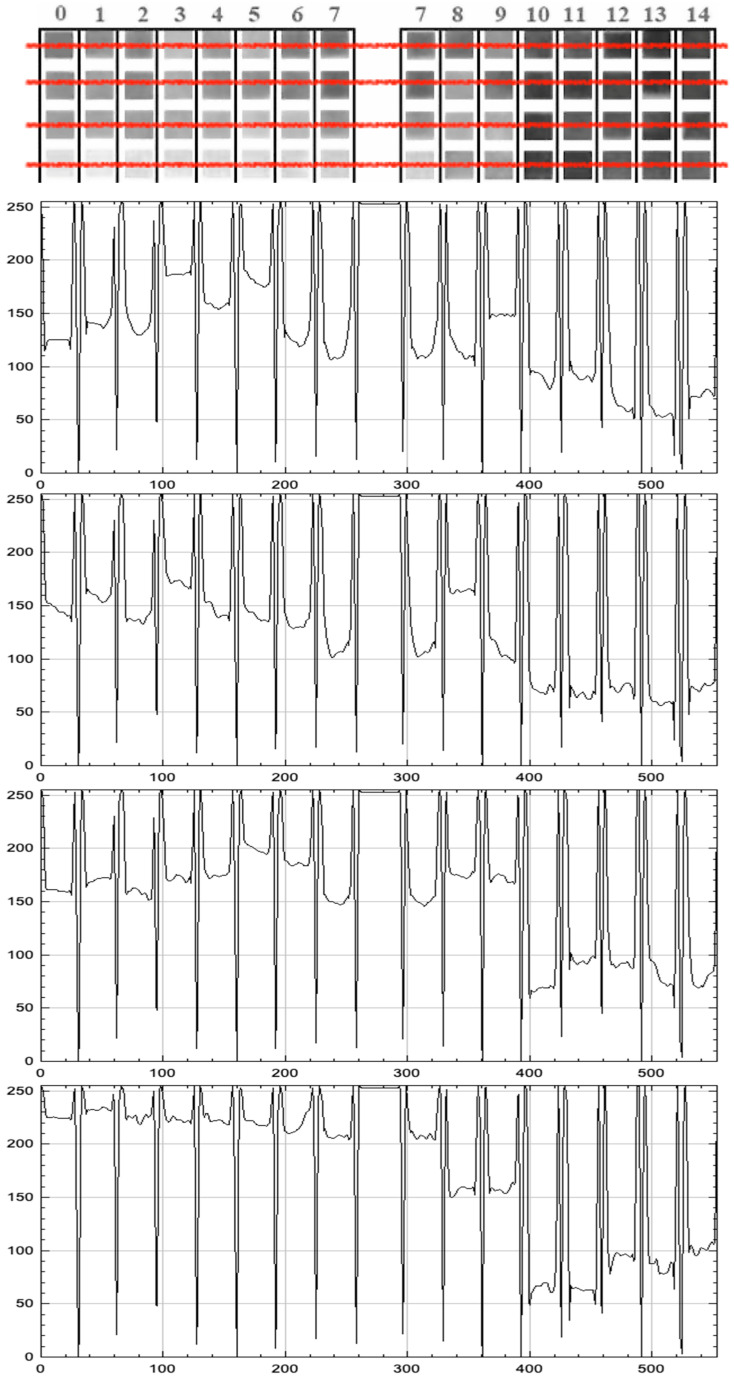
Grayscale line-scan (horizontal red line) intensity profiles for four dye-based pH strips exposed to buffers from pH 0 to 14. Panels from top to bottom: *Hibiscus*, *Purple cabbage*, *Radish*, *Turmeric*. Each curve shows pixel intensity (0–255 arbitrary units) along the horizontal position; vertical minima mark inter-pad gaps. The thumbnail mosaics above each plot depict the corresponding pad sequence (0–14).

**Figure 10:**
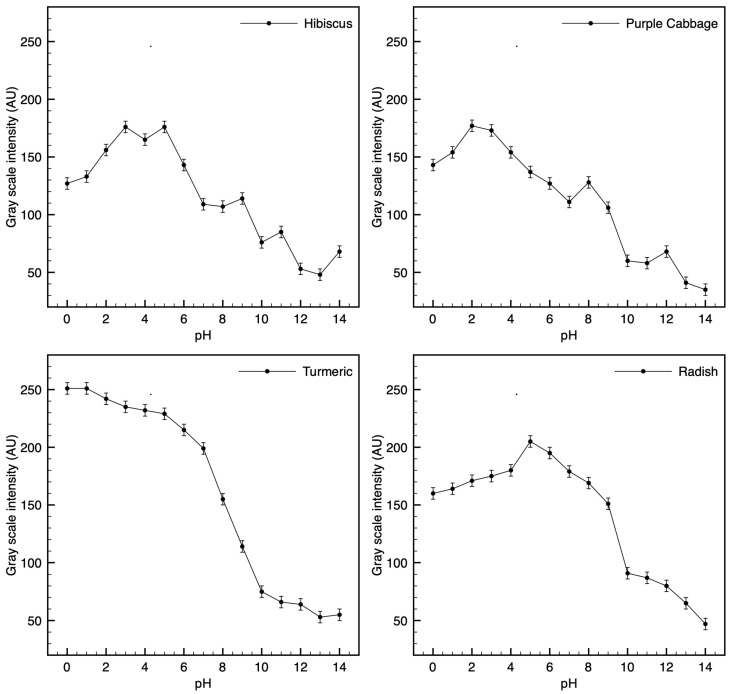
Grayscale intensity versus pH (0–14) for each dye: Hibiscus, Purple cabbage, Turmeric, and Radish. Curves show the mean pixel intensity (arbitrary units) with error bars indicating the standard deviation from (*n* = 3) replicate pads per pH. Non-monotonic segments reflect dye-specific hue changes captured in grayscale.

The resulting profiles showed a partial correlation between pH and grayscale intensity, enabling semi-quantitative interpretation in selected pH ranges (see Table 3 and Figure 8):

The grayscale response was non-linear and pigment-dependent. For example, turmeric exhibited a consistent intensity gradient across all pH values, indicating potential applications in digital or smartphone-assisted sensing platforms. In contrast, hibiscus and radish exhibited plateaus in low-pH regions, limiting their application in highly acidic environments (see Figure 9 and Figure 10).

Despite the promise of digital analysis, some limitations remain. Variability in light conditions, sensor sensitivity, and paper texture can affect accuracy. Therefore, visual inspection remains the most effective and accessible method for using these indicators, especially in field settings without image-processing tools.

### 3.4. Comparison and Application Potential

The four selected pigments were each able to indicate a wide span of pH, but with individual strengths as noted. Summarizing the performance:

- Turmeric was the most versatile and robust pigment, showing a clear color and brightness change across the full pH 0–14 range. It maintained a yellow-to-brown gradation that could potentially allow continuous estimation.
- Purple cabbage and hibiscus offered excellent color contrast in the neutral-to-alkaline pH range (they turn distinctly different colors above pH ~6), making them suitable for applications like food packaging or environmental water testing where neutral-to-basic pH changes are relevant. However, in very acidic conditions (pH < 3), both tended to simply be bright red, limiting their distinguishability in that range.
- Radish performed best in mid-to-high pH levels (it became a strong purple or gray above pH 8) but is less reliable at pH levels below 5 (little visible change in the red-pink region). It could be useful for detecting the onset of alkalinity, but not for differentiating strong acids.

**Figure 11:**
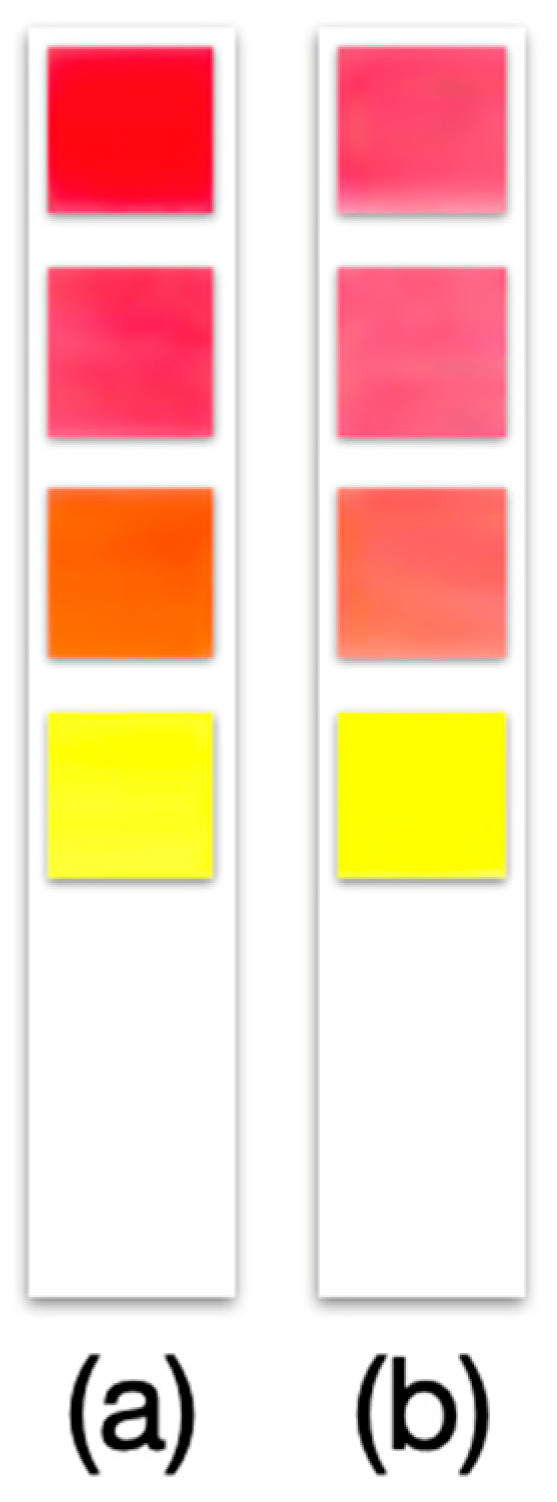
Prototype of pH test strip assembled with four 50% cotton:50% cellulose papers of 5 × 5 mm impregnated with natural organic dye. (**a**) Freshly manufactured and (**b**) after 30 days of storage at 22 ± 5 °C, humidity 30 ± 5%, and light and darkness cycles of 12 h/12 h.

Significantly, all four pigments demonstrated sufficient stability on the paper substrate, maintaining their color and response properties after drying and storage under ambient conditions for at least 30 days. This short-term stability test (up to one month) suggests that the strips do not immediately degrade and can be prepared in advance and used when needed. The prototype strips (kept in closed containers, in the dark, at room temperature) showed no visible color loss or shift over 15 and even 30 days, and the pH response on Day 30 was essentially the same as initially observed. (Figure 11 shows a prototype strip immediately after fabrication and another after 30 days of storage, with no obvious difference in color.) In future work, a comparison will be made between the stability of commercial dyes and that of organic dyes; however, this was not one of the initial objectives of the work. Nevertheless, it is not ruled out for future work to strengthen the research.

## 4. Discussion

This study demonstrates the feasibility of paper-based colorimetric pH test strips fabricated with bio-derived dyes (hibiscus, purple cabbage, radish, turmeric). Across pH 0–14, the four systems produced clear hue/intensity changes, with turmeric showing detectable contrast across the full range, while anthocyanin-rich dyes (hibiscus, purple cabbage, radish) exhibited reduced contrast at very low pH. These observations support the intuitive use of plant dyes for affordable pH indication and provide a basis for semi-quantitative readout using image analysis. In particular, turmeric demonstrated full-spectrum responsiveness, confirming its potential as a broad-spectrum natural indicator [16,34,35].

Digital image analysis provided semi-quantitative insights into pigment behavior, revealing that grayscale intensity profiles can partially correlate with pH values in specific ranges. For example, hibiscus and radish allowed grayscale-based detection within the pH range of 5 to 14. At the same time, purple cabbage was detected between pH 3 and 14, and turmeric was detected across the entire pH range of 0 to 14, demonstrating its robustness and reliability [20,25].

The grayscale analysis was chosen rather than an analysis in the color space because (i) it simplifies the processing pipeline, (ii) it is less sensitive to small color-balance shifts than individual RGB channels, and (iii) our device is primarily intended for visual, low-resource applications. We are considering studying it under an experimental design made for that purpose.

However, it is essential to emphasize that, despite the utility of grayscale analysis, visual inspection remains the most effective and accessible method for determining pH using these natural indicators. Human perception excels in detecting complex color gradients, particularly when variability in lighting or image resolution limits digital interpretation [12,30]. This makes these indicators especially suitable for use in educational settings, environmental fieldwork, and low-resource communities, where instrumentation may not be available.

In agreement with prior research on anthocyanins and curcuminoids [12,14,16,27], the findings confirm that plant-based pigments are highly responsive to pH changes, exhibiting sufficient stability under ambient conditions when properly processed and applied. Moreover, the ability to produce test strips using non-toxic, biodegradable materials aligns with the global movement toward greener chemical technologies and eco-friendly analytical tools [36,37].

## 5. Conclusions and Future Work

This project demonstrates the feasibility and practicality of developing natural pH indicators from plant-derived pigments, offering a sustainable and low-cost alternative to synthetic acid-base indicators. Through a systematic screening of 40 plant species based on availability and extractability, four pigments—Hibiscus sabdariffa, *Brassica oleracea* var. *capitata f. rubra* (purple cabbage), *Raphanus sativus* (radish), and *Curcuma longa* (turmeric)—were selected for their distinct and reproducible chromatic responses to a wide range of pH values.

These findings support the development of eco-friendly pH test sensors based on plant-derived colorants. Their combination of low cost, biodegradability, and acceptable stability positions them as practical tools for educational, diagnostic, and environmental monitoring applications.

One limitation of field quantitation is that the illumination and camera settings were controlled but not fully standardized across all acquisitions, even with a color reference; small variations in white balance or illuminance (lux) can bias grayscale intensity. Additionally, we did not systematically assess interferences (ionic strength, divalent cations, sugars, proteins, amines) at fixed pH levels. In this sense, the immediate future work should focus on (i) improving long-term stability through encapsulation techniques, (ii) enhancing digital interpretation via calibrated colorimetry and machine learning algorithms applied to RGB/HSV image data, and (iii) exploring combinatorial pigment systems for extended sensitivity and color gradient refinement.

Finally, this research underscores the integration of natural pigments into portable, accessible, and sustainable pH-sensing platforms, thereby bridging traditional knowledge and modern materials science for a diverse range of practical applications.

## Figures and Tables

**Table 1:** Plant species screened as potential natural pH indicators, showing scientific and common names and the plant part (leaf, fruit, or peel) used for pigment extraction.

Image Number	Scientific Name	Common Name	Leaf	Fruit	Peel
1	*Nephlium lappaceum* L.	Rambutan			*
2	*Capsicum pubescens*	Rocoto Pepper		*	
3	*Musa paradisiaca* L.	Banana			*
4	*Theobroma cacao* L.	Cocoa Seeds		*	
5	*Coffea arabica* L.	Coffee		*	
6	*Cinnamomum verum* J.	Cinnamon	*		
7	*Prunus serotina*	Black Cherry			*
8	*Margonodiade*	Cochineal carmine			*
9	*Allium cepa* L.	Onion		*	
10	*Prunus avium* L.	Cherry Fruits		*	
11	*Amaranthus hybridus* L.	Amaranth Leaves	*		
12	*Spinacia oleracea* L.	Spinach	*		
13	*Eucalyptus globulus* Labill.	Eucalyptus	*		
14	*Bougainvillea glabra* Choisy	Bougainvillea Flower	*		
15	*Hibiscus sabdariffa* L.	Hibiscus/Roselle Flower	*		
16	*Tabebuia chrysantha*	Yellow Trumpet Tree Flower	*		
17	*Handroanthus impetiginosus*	Pink Trumpet Tree Flower	*		
18	*Psidium guajava* L.	Guava		*	
19	*Laurus nobilis* L.	Bay Laurel	*		
20	*Citrus reticulata*	Mandarin Orange			*
21	*Mangifera indica* L.	Red Mango			*
22	*Malus domestica*	Red Apple			*
23	*Rubus glaucus* Benth.	Andean Blackberry		*	
24	*Vaccinium floribundum* Kunth	Andean Blueberry		*	
25	*Solanum quitoense* Lam.	Naranjilla			*
26	*Juglans neotropica* Diels	Andean Walnut	*		
27	*Capsicum annuum* L. var. *grossum*	Yellow Bell Pepper		*	
28	*Capsicum annuum* L. var. *grossum*	Orange Bell Pepper		*	
29	*Ananas comosus*	Pineapple			*
30	*Selenicereus megalanthus*	Yellow Dragon Fruit			*
31	*Hylocereus costaricensis*	Red Dragon Fruit			*
32	*Raphanus sativus* L.	Radish			*
33	*Prunus domestica* L.	Greengage Plum			*
34	*Beta vulgaris* L.	Red Beet		*	
35	*Hibiscus rosa-sinensis* L.	Chinese Hibiscus	*		
36	*Croton lechleri* Müll. Arg.	Dragon’s Blood Tree Resin		*	
37	*Solanum betaceum* Cav.	Tamarillo/Tree Tomato			*
38	*Daucus carota* subsp. *sativus*	Carrot		*	
39	*Brassica oleracea* var. *capitata f. rubra*	Red Cabbage	*		
40	*Citrus x aurantiifolia*	Lime			*

The asterisk (*) denotes the plant part used for extract preparation (leaf, fruit, or peel).

**Table 2:** Pigments selected and evaluated as pH indicators, and the nominal pH range over which a visible color change was observed.

Colorant	pH Range with Visible Color Shift
Hibiscus	0–14
Purple Cabbage	0–14
Radish	0–14
Turmeric	0–14

**Table 3:** Dyes selected for the pH test strips and the pH range over which their color response can be reliably distinguished by grayscale image analysis.

Colorant	Reliable pH Detection via Grayscale
Hibiscus	pH 0–14
Purple Cabbage	pH 0–14
Radish	pH 0–14
Turmeric	pH 0–14

## Data Availability

The original contributions presented in the study are included in the article, and further inquiries can be directed to the corresponding authors.

## Abbreviations

The following abbreviations are used in this manuscript:

pH	Potential hydrogen
LED	Light Emitting Diode
